# Acute adrenal insufficiency secondary to bilateral adrenal B-cell lymphoma: a case report and review of the literature

**DOI:** 10.3332/ecancer.2016.634

**Published:** 2016-04-18

**Authors:** Carlos De Miguel Sánchez, Luis Ruiz, Jose Luis González, Jose Luis Hernández

**Affiliations:** 1 Haematology Division, Hospital Universitario de Álava, Vitoria 01009, Spain; 2 Cardiology Division, Hospital Marqués de Valdecilla-IDIVAL, University of Cantabria, Santander 39008, Spain; 3 Department of Internal Medicine, Hospital Marqués de Valdecilla-IDIVAL, University of Cantabria, Santander 39008, Spain

**Keywords:** primary adrenal lymphoma, adrenal insufficiency, non-Hodgkin lymphoma

## Abstract

Primary adrenal lymphoma is an extremely rare entity which constitutes less than 1% of extranodal lymphomas. Most cases present with bilateral adrenal masses and without extraadrenal involvement, which can lead to symptoms of adrenal insufficiency. The prognosis is usually poor and chemotherapy is the first-line treatment option. We report here on a 78-year-old man admitted to our Internal Medicine Department because of constitutional symptoms and high fever spikes. He was diagnosed with adrenal insufficiency and a CT-scan revealed bilateral adrenal masses of about 6 cm in diameter. A percutaneous biopsy was performed and the histological exam was consistent with diffuse large B cell lymphoma. A review of the literature of this unusual entity was also carried out.

## Introduction

Primary extranodal lymphomas represent approximately one third of all lymph node neoplasms. Moreover, non-Hodgkin lymphomas that arise from endocrine organs are very infrequent, accounting for only 3% of extranodal lymphomas with the thyroid being the organ most affected [1]. Primary adrenal lymphoma (PAL) constitutes less than 1% of extranodal lymphomas and in nearly 70% of cases they are bilateral. In these cases, the development of adrenal insufficiency is more frequent [2–5]. The most common subtype of PAL is diffuse large B cell lymphoma, which comprises more than 70% of the cases. The prognosis is usually poor and chemotherapy is the first-line treatment option. We here report a patient with adrenal insufficiency secondary to bilateral involvement of adrenal glands by a large cell lymphoma. A review of the literature on the most relevant clinical aspects of the PAL is also presented.

## Case report

A 78-year-old man was admitted to our Internal Medicine Department because of asthaenia, anorexia, unexplained weight loss (6 kg in the last four months), and high fever spikes lasting for two weeks. His past medical history includes hypertension, type 2 diabetes, hypercholesterolemia, and a transient ischemic attack. Two months before admission he underwent stent placement in the right coronary and circumflex arteries because of ischemic heart disease.

On admission, his blood pressure was 100/60 mmHg and his temperature 38.2°C. There were no enlarged lymph nodes, hepatosplenomegaly, or other positive physical examination findings. A full blood count was normal and the erythrocyte sedimentation rate was 23 mm/hour. A biochemical profile showed potassium 8.1 mEq/L, sodium 128 mEq/L, serum creatinine 2.3 mg/dL, C-reactive protein 11.2 mg/dL, LDH 1383 IU/L, and ferritin 7275 mg/dL.

**Figure 1.**
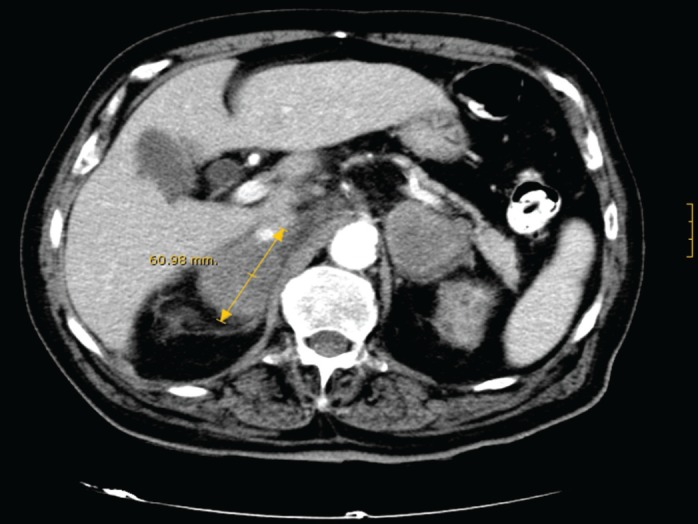
CT-scan showing a bilateral heterogeneous mass of about 6 cm in diameter in both adrenal glands.

With the suspicion of adrenal insufficiency, he was started on hydrocortisone and fludrocortisone, with progressive clinical improvement and correction of hyponatremia, hyperkalemia, and renal function. During admission he continued to be febrile with spikes of up to 39°C, which resolved with oral naproxen. Baseline serum cortisol levels were 13 mg/dl, with no response to ACTH stimulation. Serum ACTH levels were 217 pg/mL (normal <50 pg/mL), and 24-hour urinary free cortisol was 13 μg (normal, 20–90 μg). An abdominal CT-scan (Figure 1) showed a heterogeneous mass of about 6 cm in diameter in each adrenal gland without other structural abnormalities or lymph node enlargement. F^18^FDG PET/CT-scan confirmed these findings, and showed high metabolic activity in both tumours, highly suggestive of malignancy.

A percutaneous Tru-cut needle biopsy of the left adrenal gland was performed. The histological examination revealed diffuse large B-cell lymphoma positive for CD20, BCL-2, BCL-6, and Ki-67 stain, showing a proliferative index >90%. Immunostains for cyclin D-1, CD10, S-100, chromogranin A, pankeratin, and calretinin were negative. Staging was completed with a bone marrow biopsy, in which no neoplastic invasion was observed. Finally, a diagnosis of large B cell extranodal lymphoma (bilateral adrenal, which determines functional impairment) IIEB stage with an international prognostic score of 3 was made.

**Figure 2.**
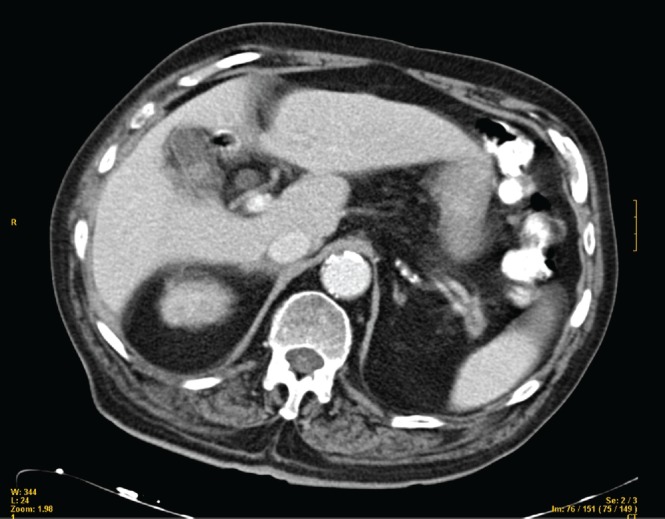
CT-scan at the end of chemotherapy treatment showing complete response.

Five cycles of chemotherapy with cyclophosphamide, doxorubicin, vincristine, prednisone, and rituximab (R-CHOP) and one cycle with cyclophosphamide, vincristine, prednisone, and rituximab (R-CVP) were administered reaching complete response (Figure 2).

The patient remained in complete remission for two years, until his death because of brain metastasis of an epidermoid lung cancer diagnosed after PAL.

## Discussion

PAL is a very rare type of extranodal lymphoma with only about 100 cases described in the medical literature [2]. The male: female ratio is 3:1 and the average age is usually around 70 years [2, 3, 4, 6]. In bilateral PAL, male: female ratio is lower (1.6:1) and the mean age at presentation is around 65 years [7].

Nowadays, the etiology of PAL remains unclear. The most common hypothesis involves haematopoietic tissue akin to adrenal myelolipoma resting in the adrenal glands. Furthermore, it is suggested that PAL arises on a background of previous autoimmune adrenalitis. Immune dysfunction, Epstein-Barr virus, and mutations in the *p53* and *c-kit* genes are also thought to be implicated in the pathogenesis of PAL [3].

Secondary infiltration of adrenal gland by non-Hodgkin’s lymphoma has been reported in 25% of cases, and usually presents as a unilateral neoplasm. Nevertheless, the rare PAL is commonly bilateral and presents as adrenal masses without any other extraadrenal involvement. Clinical features are typically nonspecific symptoms, such as asthaenia, weight loss, vague abdominal pain or fever, and therefore an early diagnosis is difficult. In half of the cases, symptoms and signs of adrenal insufficiency secondary to bilateral infiltration of the adrenal glands may be present. However, unilateral PAL does not exhibit manifestations (whether clinical or laboratory data) of glandular failure [2, 8]. Development of Addisonian crisis can lead to severe life-threatening consequences, therefore immediate substitution therapy must be considered if PAL is suspected.

High-grade diffuse large B-cell lymphoma is the predominant histological subtype of PAL (78% of cases), while a T-immunophenotype has only been reported in approximately 10% of the cases [8–11]. It is important to make a proper differential diagnosis, including other diseases that can affect simultaneously both adrenal glands, such as tuberculosis, histiocytosis, pheochromocytoma, or metastatic spread of solid tumours [10].

Regarding diagnosis, percutaneous CT or ultrasound-guided biopsy is the procedure of choice. F^18^FDG PET imaging can be a useful tool especially for staging the disease [8]. Immunohistochemical studies have important implications for diagnosis, management, and more comprehensive prognosis of these neoplasms especially some markers such as Bcl-2, CD20, and Ki67.

At present chemotherapy is the cornerstone in the management of PAL, mainly the classical regimens (CHOP) with the addition of rituximab, and clinical responses have been achieved mainly in patients with early stages [7, 12]. It has been proposed using laparoscopic surgery as adjuvant to chemotherapy for masses larger than 6 cm [2]. The role of radiation therapy in PAL is unclear, and the potential benefits of radiation need to be weighed against its negative effects on adrenal function [11]. Similarly, the role of autologous peripheral blood stem cell transplantation is also unclear, and its use should be individualised. It is being considered a possible therapeutic approach in young patients, and in the same line some authors have reported encouraging results with this therapeutic option [13, 14]. Because of life-threatening consequences of adrenal insufficiency, glucocorticoid replacement therapy might be necessary [14]. Nevertheless, prognosis of PAL is worse than the other extranodal lymphomas, with the median survival being around 12 months [7]. Several factors have been associated with a poor prognosis, such as the expression of Bcl-2 protein and Epstein-Barr virus infection (especially in NK and T cell subtypes). The wide systemic spread of the tumour, including gastric and central nervous system invasion, has been reported.

In conclusion, PAL should be included in the differential diagnosis of patients presenting with bilateral adrenal masses.

## Conclusion

PAL is an extremely unusual extranodal lymphoproliferative entity which most commonly affects elderly men. In most cases PAL presents with bilateral adrenal masses without any other extraadrenal involvement, and in approximately 50% of patients, symptoms of adrenal insufficiency may be present. High-grade diffuse large B-cell lymphoma is the predominant histological subtype of this entity. The prognosis of primary bilateral adrenal lymphoma is usually poor and chemotherapy is the first-line treatment option. PAL should be considered in the differential diagnosis of patients presenting with bilateral adrenal masses without nodal or extranodal involvement.

## Conflicts of interest

The authors declare that there are no conflicts of interest.

## Authors’ contributions

All the authors participated in the bibliography search. CDM was in charge of producing the text. All the authors approved the final version of the document.
